# Different forms of decision-making involve changes in the synaptic strength of the thalamic, hippocampal, and amygdalar afferents to the medial prefrontal cortex

**DOI:** 10.3389/fnbeh.2015.00007

**Published:** 2015-01-30

**Authors:** Juan Carlos López-Ramos, Rafael Guerra-Narbona, José M. Delgado-García

**Affiliations:** Division of Neurosciences, Pablo de Olavide UniversitySeville, Spain

**Keywords:** decision making, medial prefrontal cortex, hippocampus, basolateral amygdala, mediodorsal thalamus, mice, field postsynaptic potentials, associative learning

## Abstract

Decision-making and other cognitive processes are assumed to take place in the prefrontal cortex. In particular, the medial prefrontal cortex (mPFC) is identified in rodents by its dense connectivity with the mediodorsal (MD) thalamus, and because of its inputs from other sites, such as hippocampus and amygdala (Amyg). The aim of this study was to find a putative relationship between the behavior of mice during the performance of decision-making tasks that involve penalties as a consequence of induced actions, and the strength of field postsynaptic potentials (fPSPs) evoked in the prefrontal cortex from its thalamic, hippocampal, and amygdalar afferents. Mice were chronically implanted with stimulating electrodes in the MD thalamus, the hippocampal CA1 area, or the basolateral amygdala (BLA), and with recording electrodes in the prelimbic/infralimbic area of the prefrontal cortex. Additional stimulating electrodes aimed at evoking negative reinforcements were implanted on the trigeminal nerve. FPSPs evoked at the mPFC from the three selected projecting areas during the food/shock decision-making task decreased in amplitude with shock intensity and animals’ avoidance of the reward. FPSPs collected during the operant task also decreased in amplitude (but that evoked by amygdalar stimulation) when lever presses were associated with a trigeminal shock. Results showed a general decrease in the strength of these potentials when animals inhibited their natural or learned appetitive behaviors, suggesting an inhibition of the prefrontal cortex in these conflicting situations.

## Introduction

When an animal has to select one out of two or more actions, a conjunction of cognitive processes is developed in order to take the most appropriate decision. If the action is aimed at obtaining a benefit, but involves a danger, a so-called risk-based decision-making has to be executed (Floresco et al., 2008; St. Onge et al., 2012). It is widely recognized, in humans and other primate and non-primate species, that the prefrontal cortex has a major role in these functional processes (Bechara et al., 2003; Bechara, 2005), although the role of the amygdala (Amyg), nucleus accumbens, or the dopamine system in this type of tasks has been proposed too.

Among the main connections of the prefrontal cortex are afferents from the hippocampal CA1 area (Thierry et al., 2000; Takita et al., 2007; Parent et al., 2010), and the two bidirectional connections with the mediodorsal (MD) thalamic nucleus (Rose and Woolsey, 1948; Herry et al., 1999; Herry and Garcia, 2002), and the Amyg (Gabbott et al., 2005, 2012; Cressman et al., 2010). In particular, hippocampal afferents innervate the whole rostro-caudal extent of the prelimbic area, although different innervations patterns can be observed within its dorsal and ventral portions. Thus, varicose fibers and terminal hippocampal arborizations are present in layers II–VI of the ventral portion, while the innervation is less dense in the deep layers (V–VI) of the dorsal prelimbic area. The hippocampal innervation is distributed in all layers of the medial orbital area, with a slight preference for III–VI layers (Thierry et al., 2000). Moreover, a discrete projection from the CA1/subiculum to the agranular insular area of the lateral prefrontal cortex, which sends collaterals to the medial prefrontal cortex (mPFC), has been also described (Verwer et al., 1997). On the other hand, axon terminals from MD have been shown to make direct synaptic contacts with apical dendrites of pyramidal neurons in layers III, V, and VI of the rat, while corticothalamic cells make asymmetrical synapses with thalamocortical neurons from MD (Kuroda et al., 1995a,b). Additionally, synaptic contacts between MD thalamocortical terminals and GABAergic interneurons have been described in the prelimbic cortex and also between GABAergic terminals and corticothalamic neurons projecting to the MD (Kuroda et al., 2004). Finally, neurons in the BLA projects monosinaptically onto corticospinal neurons in the mPFC that, at the same time, projects to the thoracic spinal cord and to subcortical regions, as the lateral hypothalamus (Gabbott et al., 2012).

All the pathways established between the hippocampal CA1 area, the MD nucleus, or the Amyg, and the mPFC (CA1-mPFC, MD-mPFC, and Amyg-mPFC, respectively) have been reported to play different and specific roles in cognitive processes such as decision-making tasks (Bechara et al., 2003; Floresco and Ghods-Sharifi, 2007; Kawagoe et al., 2007; Floresco et al., 2008; St. Onge et al., 2012; Calhoon and O’Donnell, 2013; Belchior et al., 2014; Yu and Frank, 2014). In most of the studies about risk-based decision making, the task consisted in choice trials in which animals had the opportunity to select between smaller certain (safer) or larger uncertain (riskier) rewards and, in them, the lesion or blockage of involved neural structures was used to determine their respective roles. Thus, Mobini et al. (2002) assessed the effects of lesions of the orbitofrontal cortex on the sensitivity to probabilistic reinforcement, concluding that these lesions can promote preference for the smaller and more certain of the two reinforcers. St. Onge et al. (2012) disrupted the bilateral connections between mPFC and amygdalar nuclei, revealing that the disruption of information transfer from the mPFC to the basolateral amygdala (BLA), but not the reciprocal, increased the choices of larger, riskier options. On the other hand, Cardinal and Howes (2005) blocked the function of the nucleus accumbens by quinolinic acid injections, concluding that this nucleus contributes to action selection by promoting the choice of uncertain, as well as delayed, reinforcements.

Due to these complex interactions between these different cortical and subcortical areas, the aim of this work was to study the role played by the MD-mPFC, CA1-mPFC, and Amyg-mPFC synapses in decision-making tasks. For this, we developed two risk-based decision making tasks in which, instead of two different reinforcements (a safer modest and a riskier larger one), the consecution of a single reward implied the risk of receiving a punishment. Thus, we have simplified the task and avoided the need of transient or permanent blockage of any of the neural structures included in this study. According to that, we have recorded in alert behaving mice the field post-synaptic potentials (fPSPs) evoked at these three synapses during the performance of three different behaviors (fleeing from the feeder, approaching the feeder, and feeding) carried out by the experimental animal in a decision-making task involving a food reward accompanied or not by an electrical shock (mild or strong) presented to the trigeminal nerve. In addition, we recorded fPSPs evoked at the same three synapses during a decision-making, operant conditioning task. In this case, fPSPs were evoked during the performance of three different behaviors (fleeing from the lever, approaching the lever, and pressing the lever). Each lever press was accompanied by a reward consisting of a pellet of food. These three behaviors could be accompanied or not by the electrical stimulation (mild or strong) of the trigeminal nerve. We also studied the electrophysiological properties of the three mentioned synapses by determining their input/output curves (I-O), paired-pulse facilitation (P-P), and long-term potentiation (LTP) through a high-frequency-stimulation (HFS) protocol. The main results collected from this study suggest a significant involvement of these three synaptic inputs to the mPFC during the performance of different behaviors related to specific decision-making tasks, as described and discussed below.

## Material and methods

### Experimental subjects

Experiments were performed on C57Bl/6 adult male mice (4–6 months old; 25–33 g) obtained from an official supplier (University of Granada Animal House, Granada, Spain). Before surgery, animals were housed in separate cages (*n* = 10 per cage). The mice were kept on a 12:12 h light-dark cycle with constant ambient temperature (21 ± 1°C) and humidity (50 ± 7%). Unless otherwise indicated, food and water were available *ad libitum*.

**Figure 1:**
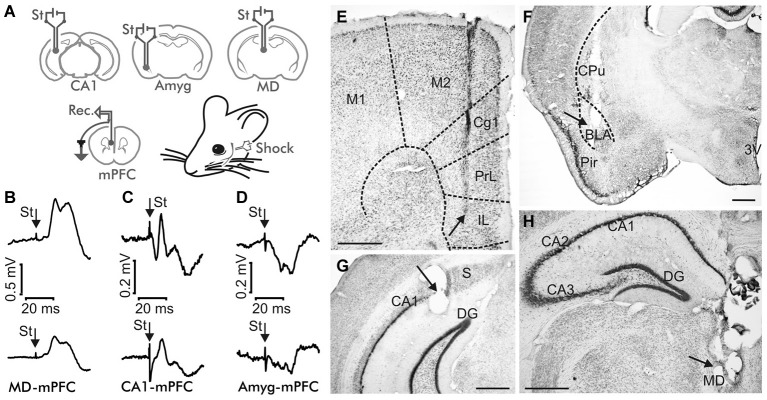
**Experimental design. (A)** Animals were implanted with bipolar stimulating (St.) electrodes in one of these three sites: the mediodorsal (MD) thalamic nucleus, the hippocampal CA1 area, or the basolateral subdivision of the amygdala (Amyg), and with recording electrodes in the medial prefrontal cortex (mPFC). **(B–D)** Average of ≥10 fPSP recordings evoked at the mPFC by stimulation of the MD thalamic nucleus **(B)**, the hippocampal CA1 **(C)** and the Basolateral Amygdala (BLA, **D**), corresponding to the 4th No Shock session (top) and to the 1st Strong Shock session (bottom) of the Decision Making Fear Conditioning experiment. St, stimulus. Calibration as indicated. Note the different calibration in (**B**). **(E–H)** Representative photomicrographs illustrating the location of a recording mPFC electrode **(E)**, and of stimulating electrodes implanted in the BLA **(F)**, the hippocampal CA1 area **(G)**, and the MD thalamic nucleus **(H)**. Abbreviations: 3V, third ventricle; Cg1, cingulate cortex; Cpu, caudate-putamen; DG, dentate gyrus; IL, infralimbic area of the cortex; M1, M2, motor cortex areas 1 and 2; Pir, piriform cortex; PrL, prelimbic area of the cortex; S, subiculum. Calibration bars: 500 μm.

**Figure 2:**
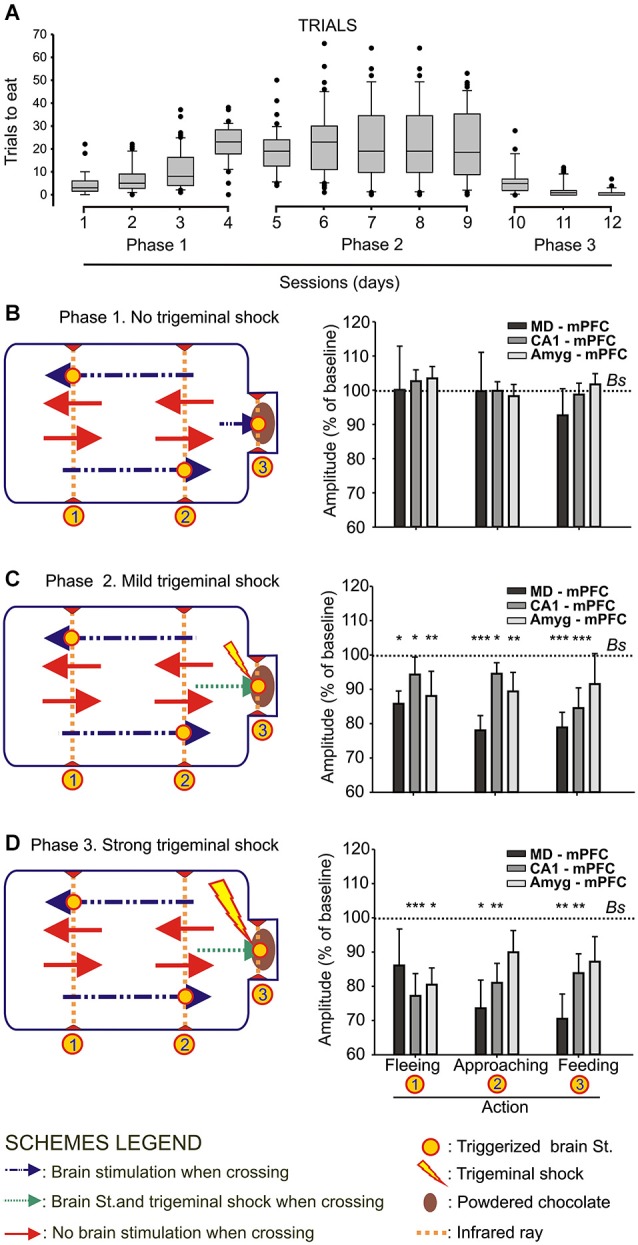
**Animal performance and changes in strength evoked at the MD-mPFC, CA1-mPFC, and Amyg-mPFC synapses during a food/shock decision-making task. (A)** On the left are shown whisker boxes summarizing the number of trials to obtain food during the first phase of the experiment: sessions 1–4, in absence of trigeminal shocks. The five middle boxes (sessions 5–9) illustrate the number of trials carried out by mice in the presence of a mild trigeminal shock (second phase). Finally, the three right boxes (sessions 10–12) illustrates the significant decrease in the number of trials in the presence of a strong trigeminal shock presented each time the animal tried to obtain food (third phase). **(B)** Changes in the amplitude of fPSPs evoked at the three selected synapses during fleeing from the feeder, approaching it, or during feeding. Baseline values were collected before the beginning of the experimental session. On the left is included a diagram illustrating the experimental design. Note that no significant differences in fPSP amplitudes were observed in any of the three synapses for the three selected behaviors when no trigeminal shocks were presented to the animals. **(C,D)** Changes in the amplitude of fPSPs evoked at the three selected synapses for the same selected behaviors when the animal received a mild **(C)** or strong **(D)** trigeminal shock on introducing its head into the feeder. The different signs used in the Figure are defined in the bottom legends. *Bs*, Baseline. Significant differences with baseline values are indicated. Data are presented as mean ± SEM, *n* ≥ 8 animals/group. *p < 0.05; **p < 0.01; ***p < 0.001. Bonferroni’s *post hoc* test. One-way ANOVA.

**Figure 3:**
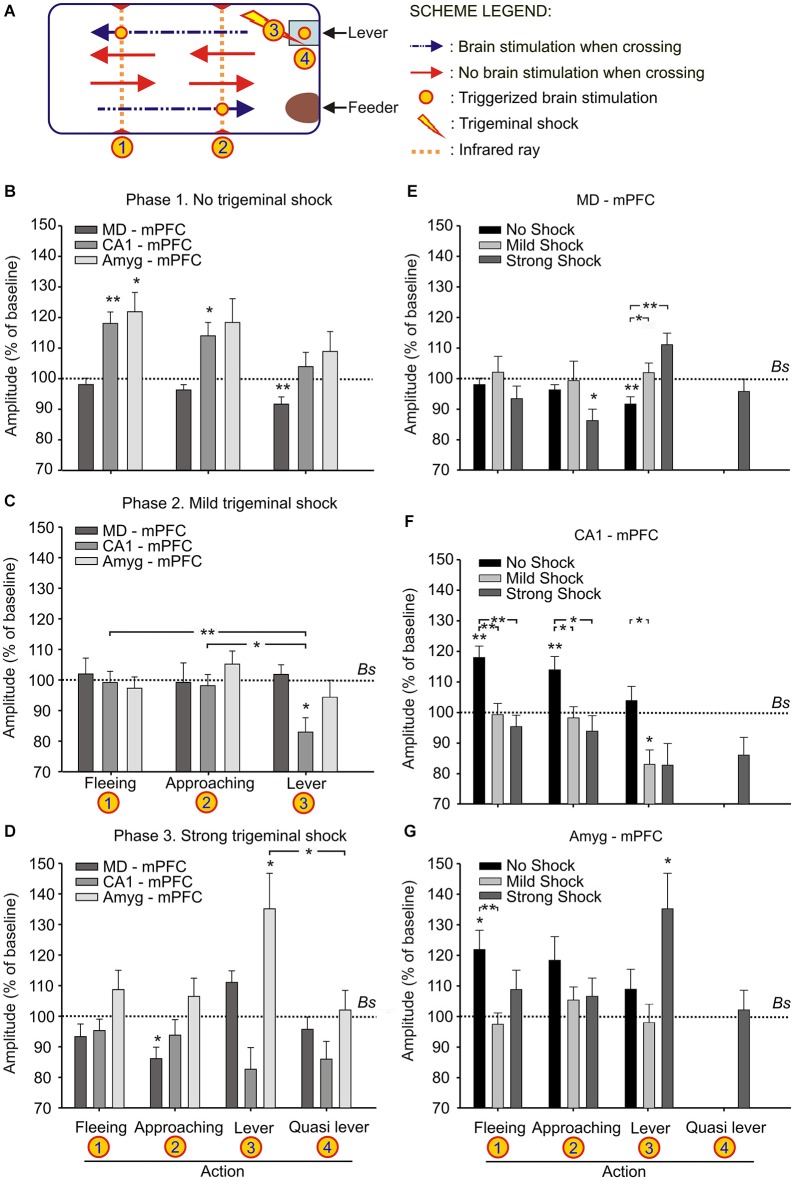
**Changes in strength evoked at the MD-mPFC, CA1-mPFC, and Amyg-mPFC synapses during a decision-making, operant conditioning task. (A)** Experimental design. We recorded fPSPs evoked at the three selected synapses during fleeing from the lever, approaching it, or during lever presses. The different signs used in the Figure are defined in the legends to the right. **(B–D)** Changes in the amplitude of fPSPs evoked at the three selected synapses during fleeing from the lever, approaching it, or during lever presses in the control situation **(B)**, and when the animal received a mild **(C)** or a strong **(D)** trigeminal shock right after pressing the lever. The “Quasi lever” columns indicate fPSP values collected when the animal was not pressing the lever, but located near it. Baseline values were collected before the beginning of the experimental session. **(E–G)** The same set of fPSP data, but organized according to the selected synapse: MD-mPFC **(E)**, CA1-mPFC **(F)**, and Amyg-mPFC **(G)**. Data are presented as mean ± SEM, *n* ≥ 8 animals/group. *p < 0.05; **p < 0.01. Comparisons were made between behaviors vs. experimental phases **(B–D)** and behaviors vs. cerebral areas **(E–G)**. *Bs*, Baseline. Asterisks outside lines compare fPSP amplitudes with baseline values (Dunnett *post hoc* test). Asterisks within lines indicate significant differences between fPSPs evoked at the same synapses during the performance of different behaviors **(C,D)** or during the performance of the same behavior in different phases of the experiment **(E–G)** (Bonferroni’s *post hoc* test). One-way ANOVA.

**Figure 4:**
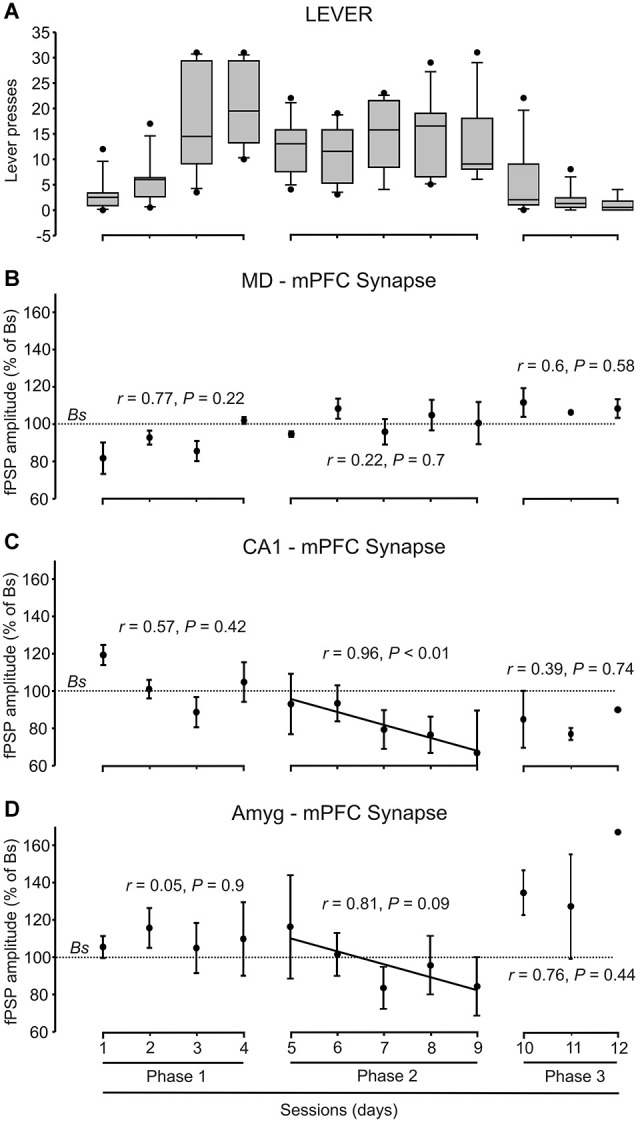
**Quantitative analysis of animal performance and fPSP data collected in the decision-making, operant conditioning task. (A)** On the left are shown the whisker boxes summarizing the number of lever presses obtained during the first phase of the Skinner box experiment: sessions 1–4 in absence of trigeminal shocks. The five middle boxes (sessions 5–9) illustrates the number of lever presses carried out by mice in the presence of a mild trigeminal shock (second phase). Finally, the three right boxes (sessions 10–12) illustrates the significant decrease in the number of lever presses in the presence of a strong trigeminal shock applied each time the animal pressed the lever (third phase). **(B–D)** Changes in the amplitude of fPSPs evoked at the MD-mPFC **(B)**, CA1-mPFC **(C)**, and AMYG-mPFC **(D)** synapses across the successive training phases and sessions during lever presses. Changes in fPSP amplitudes are with respect to the corresponding baseline (*Bs*) values. Correlation of coefficient (*r*) and statistical significance (*P*) are indicated for each phase, and a regression line is drawn when proceed.

**Figure 5:**
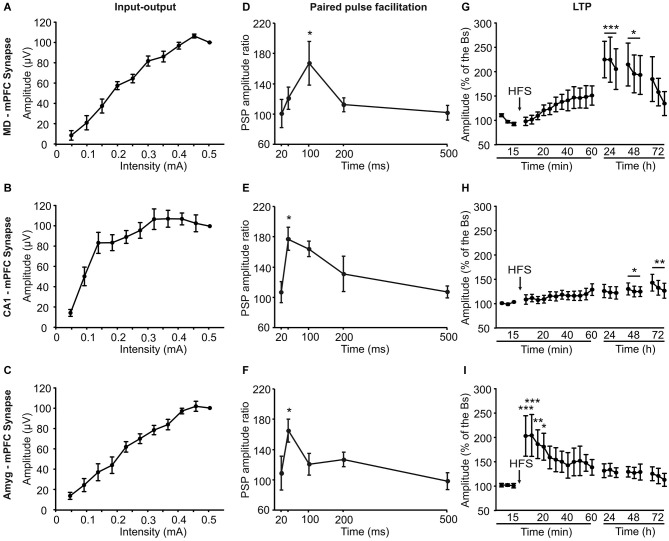
**Electrophysiological properties of the three (MD-mPFC, CA1-mPFC, and Amyg-mPFC) synapses studied in alert behaving mice**. Input-output curves **(A–C)**, paired-pulse facilitation **(D–F)**, which ratio between amplitudes was calculated through the formula [2nd/1st] × 100], and long-term potentiation (LTP; **G–I**) evoked at the three (MD-mPFC, CA1-mPFC, and Amyg-mPFC) synapses. The LTP was evoked by a high-frequency stimulation session presented at the indicated time (arrow). Note the different profiles of input-output curves, paired-pulse facilitation, and LTP evoked at the three synapses. *Bs*, baseline. Data are presented as mean ± SEM, *n* ≥ 8 animals/group. *p < 0.05; **p < 0.01; ***p < 0.001.

Animals were prepared for the chronic recording of fPSPs evoked at three different (MD-mPFC, CA1-mPFC, and Amyg-mPFC) synapses (Figure 1) during two different decision-making tasks (Figures 2–4) and during an *in vivo* study of the electrophysiological properties (I-O curves, P-P facilitation, and LTP) of the three selected synapses (Figure 5). Only animals that reached all behavioral criteria (for decision-making, operant conditioning task) and with proper electrode placement and expected field potential recordings were further used. We considered successful those animals that finished the experimental protocols presenting extracellular recordings (i.e., fPSPs) that did not deteriorate over time. A minimum of eight animals per group (i.e., per selected synapse) were used for each of the decision-making tasks. In addition, ≥10 animals/selected synapse were used for I-O curves, P-P facilitation, and the LTP study. In accordance, a grand total of 85 mice were used in this study.

Electrophysiological and behavioral studies were carried out in accordance with the guidelines of the European Union Council (2010/63/EU) and Spanish regulations (BOE 34/11370-421, 2013) for the use of laboratory animals in chronic experiments. Experiments were also approved by the local Ethics Committee (Permit Number 01/2011) of the Pablo de Olavide University (Seville, Spain).

### Surgery

Animals were anesthetized with a mixture of Ketamine (35 mg/kg) and Xylazine (2 mg/kg) i.p. Mice were prepared for the activation of three different brain synapses: (i) a first group of mice were implanted with bipolar stimulating electrodes in the right MD thalamic nucleus (0.4 mm lateral, 1.94 mm posterior, and 3.12 mm below bregma) (Paxinos and Franklin, 2001) and with a recording electrode in the ipsilateral prelimbic/infralimbic area of the mPFC (0.3 mm lateral and 1.94 mm anterior, and 3.12 mm below bregma); (ii) a second group of mice were implanted with stimulating electrodes in the right hippocampal CA1 pyramidal layer 2.75 mm lateral and 3.16 mm posterior, and 2 mm below bregma) and with a recording electrode in the indicated mPFC area; and (iii) a third group of mice were implanted with stimulating electrodes in the right Amyg (2.87 lateral, 1.82 posterior, and 4.7 below bregma) and with a recording electrode in the same mPFC area. Animals were also implanted with stimulating electrodes on the infraorbitary branch of the trigeminal nerve. These electrodes were made from 50 μm, Teflon-coated, stainless steel wire (Advent Research, Eynsham, UK). As the coating thickness was 12.5 μm, the distance between stimulating electrodes was ≥25 μm. The exposed tip was ≈300 μm in length. A bare silver wire was affixed to the bone as ground. Implanted wires were soldered to a six-pin socket (RS Amidata, Madrid, Spain) which was fixed to the skull with dental cement (Figure 1). After surgery, animals were housed in separate cages across the whole experiment. Further details of this chronic preparation can be found elsewhere (Gruart et al., 2006).

### Recording and stimulating procedures

FPSP recordings were carried out with the help of Grass P511 differential amplifiers within a bandwidth of 0.1 Hz–3 kHz (Grass-Telefactor, West Warwick, RI, USA). For the electrical stimulation of the MD, CA1, or Amyg areas during animal training, we used a 50 μs double (positive-negative) pulse at 1/3 of the intensity (in μA) necessary to evoke a maximum fPSP. The infraorbital nerve was stimulated with pulses of 500 μs and 0.5–1 mA (mild shock) or 5 ms and 0.5–1 mA (strong shock) when needed. Electrical stimulation was carried out across ISU-220 isolation units controlled by a Cibertec CS-20 dual stimulator (Cibertec, S.A., Madrid, Spain).

For I-O curves (Figures 5A–C), animals were stimulated with single pulses at increasing intensities (0.05–0.5 mA, in steps of 0.05 mA). We also checked the effects of paired pulses at different (20, 40, 100, 200, and 500 ms) inter-stimulus intervals while using intensities corresponding to 40% of the level necessary to evoke a saturating response (Figures 5D–F). For LTP induction in behaving mice we followed procedures described previously (Gruart et al., 2006; Madroñal et al., 2007; Jurado-Parras et al., 2012). FPSP baseline values (Figures 5G–I) were collected 15 min prior to LTP induction using single 50 μs, square, biphasic pulses. Pulse intensity was set at 30–40% of the level necessary to evoke a maximum fPSP response (0.05–0.25 mA)—i.e., well below the threshold for evoking a population spike. For LTP induction, animals were presented with an HFS session. This consisted of five 200 Hz, 100 ms trains of pulses at a rate of 1/s repeated 6 times, at intervals of 1 min. Thus, a total of 600 pulses were presented during the HFS session. In order to avoid evoking large population spikes and/or the appearance of EEG seizures, the stimulus intensity during HFS was set at the same as that used for generating baseline recordings. After each HFS session, the same single stimuli were presented every 20 s for 60 min. Three additional 15 min recording sessions were carried out on the following three days (Figures 5G–I).

### Food/shock decision-making task

For this task, the animal was located in a modified mouse box (25 × 13 × 10 cm; Med Associates, Inc., St. Albans, VT, USA). The box was housed within a sound-attenuating chamber (90 × 55 × 60 cm), which was constantly illuminated (19 W lamp) and exposed to a 45 dB white noise (Cibertec, S.A., Madrid, Spain). The box was equipped with a feeder located at one of its ends. The feeder was provided with chocolate powder. The box was also equipped with three infrared rays located at the sites (1, 2, 3) indicated in Figures 2B–D. When the animal crossed two rays successively, either in the direction (1 → 2) of the feeder or in the opposite (2 → 1) direction, it triggered an electrical stimulation (see above) of the implanted (MD-mPFC, CA1-mPFC, or Amyg-mPFC) synapse. In addition, when the animal introduced its head into the feeder receptacle it could receive a stimulus at the implanted synapse as well as a mild (Figure 2A) or strong (Figure 2C) trigeminal shock 500 ms later. These stimuli ranged between 0.5–2 mA, in function of the startle reaction observed previously to the beginning of the experiment, and lasted 500 μs (mild) or 5 ms (strong). Before training, mice were handled daily for 7 days and food-deprived to 80–85% of their free-feeding weight.

The experiment was carried out in three successive phases, lasting 4, 5 and 3 sessions each one, in this order and in 12 successive days. During Phase 1 (Figure 2B) the animal was introduced into the box for 20 min. This phase was repeated for four sessions in each animal. The animal was stimulated when approaching (1 → 2) the feeder or when moving away or fleeing (2 → 1) from it. No trigeminal shocks were applied during this phase, but the animal was also stimulated at the selected synapse when putting its head inside the feeder (3, Figure 2B). A maximum of 40 stimuli were presented to the animal during each session, resulting from the sum of the stimuli triggered for the crossing of the three infrared lights. In order to establish an fPSP baseline, the animal was stimulated 10 times at the selected synapse before the beginning of the session, but with the animal already located in the experimental box. Those stimuli were presented at intervals >15 s.

During the second phase, and once the animal was habituated to approach and put its head into the feeder to reach the chocolate powder (Phase 2, Figure 2C), the animal received a mild trigeminal shock (see above) when introducing its head into the feeder to obtain the chocolate powder. In this situation, the animal has to decide about the convenience of trying to eat the chocolate or not. The rest of the experiment was similar to procedures described for the first phase. A total of five recording sessions were carried out during this phase.

Finally, during the third phase (Phase 3, Figure 2D), the animal received a strong trigeminal shock (see above) when introducing its head into the feeder. The rest of the experiment was similar to the above description. A total of three recording sessions were carried out in this phase.

FPSPs evoked during fleeing, approaching, or feeding were recorded and stored during the successive sessions of the three experimental phases for off-line analysis.

### Decision-making, operant conditioning task

For this experiment, the animal was located in a Skinner box module measuring 12.5 × 13.5 × 18.5 cm (Med Associates, Inc.). Each Skinner box was housed within a sound-attenuating chamber (90 × 55 × 60 cm; Cibertec, S.A.), which was constantly illuminated (19 W lamp) and exposed to a 45 dB white noise. The Skinner box was equipped with a food dispenser from which pellets (MLabRodent Tablet, 20 mg; Test Diet, Richmond, IN, USA) could be delivered by pressing a lever. The Skinner box was also equipped with three infrared lights located at the sites (1, 2, 3) indicated in Figure 3A. When the animal crossed two lights successively, either in the direction (1 → 2) of the feeder or in the opposite (2 → 1) direction, it triggered an electrical stimulation (see above) of the implanted (MD-mPFC, CA1-mPFC, or Amyg-mPFC) synapse. In addition, when the animal pressed the lever it received an additional stimulus at the implanted synapse and it could receive a mild or a strong trigeminal shock (Figure 3A). Before training, mice were handled daily for 7 days and food-deprived to 80–85% of their free-feeding weight.

In an initial phase of this experiment, animals were trained in the Skinner box with a fixed-ratio (1:1 FR) paradigm until they learned to press the lever once in order to obtain a small pellet of food. When the initial criterion was reached (pressing the lever 15 times during two successive 20 min sessions) the experiment proper was started. The experiment was divided in three successive phases.

During the first phase (Figure 3A) the animal was placed in the Skinner box for 20 min. This phase was repeated for four sessions in each animal. The animal was stimulated when approaching (1 → 2) the lever or when moving away or fleeing (2 → 1) from it. No trigeminal shocks were applied during this phase, but the animal was also stimulated at the selected synapse when pressing the lever (3, Figure 3A). A maximum of 40 stimuli were presented to the animal during each session. In order to establish an fPSP baseline, the animal was stimulated 10 times at the selected synapse before the beginning of the session, but with the animal already in the experimental box. Those stimuli were presented at intervals of 15 s.

During the second phase, and once the animal was conditioned to press the lever to obtain a pellet (Figure 3A), the animal received a mild trigeminal shock (see above) just after pressing the lever, what made it to decide about the convenience to press the lever or not. The rest of the experiment was similar to procedures described for the first phase. A total of five recording sessions were carried out during this phase.

During the third phase (Figure 3A), the animal received a strong trigeminal shock (see above) immediately after pressing the lever. The rest of the experiment was similar to the above description. A total of three recording sessions were carried out in this phase.

Finally, during this third phase (Figure 3A), in which the animal hardly ever pressed the lever, it received an activation of the selected synapse, not when pressing the lever, but when located near it, and making an attempt to press it (see “quasi lever”, Figures 3D,G). FPSPs evoked during approaching the lever, pressing it, or fleeing from it were recorded and stored during the successive sessions of the three experimental phases for off-line analysis.

### Histology

Once experiments were finished, mice were deeply re-anesthetized (chloral hydrate, 400 mg/kg) and perfused transcardially with saline and 4% 0.1 M phosphate-buffered paraformaldehyde. Small electrolytic lesions (20 μA, 20 s) were carried out at the stimulating sites to facilitate their proper location. Their brains were removed, postfixed overnight at 4°C, and cryoprotected in 30% sucrose in 0.1 M PB. Sections were obtained in a cryotome (Leica, Wetzlar, Germany) at 50 μm. Selected sections including the implanted sites (prefrontal and hippocampal cortices, thalamus, amygdala) were mounted on gelatinized glass slides and stained using the Nissl technique with 0.1% toluidine blue to determine the location of stimulating and recording electrodes.

### Data collection and analysis

FPSPs, and 1-volt rectangular pulses corresponding to infrared light crosses, lever presses, and brain stimulations, were stored digitally, at a sampling rate of 1 kHz, on a computer through an analog/digital converter (CED 1401 Plus, Cambridge, England). Data were analyzed off-line for quantification of each animal’s performance in the open corridor and Skinner box, and fPSPs, using the Signal 3 (CED) program. The amplitude of the first component of the evoked fPSPs was computed in mV. FPSPs collected during baseline sessions, infrared-light crossings, and lever pressings from each session and animal were recorded and averaged. In each case, the mean value of the amplitude of the first fPSP component was determined, being the maximum amplitude latencies of 13.1 ± 1.2, 15.4 ± 1.3, and 14.3 ± 1 ms, for the MD-mPFC, CA1-mPFC and Amyg-mPFC synapses, respectively. Computed results were processed for statistical analysis using the IBM SPSS Statistics 18.0 (IBM, Armonk, New York, USA) and the Sigma Plot 11.0 (Systat Software Inc., San José, CA, USA) programs.

Data are always represented as the mean ± SEM. Statistical significance of differences between the analyzed data was inferred by one-way ANOVA and ANOVA for repeated measures (data by groups), with multiple comparisons (Bonferroni’s or Dunnet’s *post hoc* test) for a further study of significant differences. Statistical significance was set at *α* < 0.05.

## Results

### Changes in fPSPs evoked at three different prefrontal afferents during a food/shock decision-making task

As illustrated in Figure 1A, wild-type mice were implanted with bipolar stimulating electrodes in a selected MD thalamic, hippocampal CA1, or amygdalar site and with a recording electrode in the mPFC. In addition, animals were implanted with a stimulating electrode on the infraorbitary branch of the trigeminal nerve. In the first series of experiments, animals (*n* ≥ 8 per selected synapse) were trained in a modified mouse box to carry out the simple task of collecting chocolate powder from a feeder located at one end of the box. As illustrated in Figure 2B, during Phase 1 of habituation in the absence of any trigeminal shock, the experimental animal was free to move in the box and to approach the feeder for a 20 min period. As indicated in Methods, the box was provided with 3 sets of infrared lights—two of them (1, 2) located at the main body of the box, and the third (3) at the feeder entrance. The animal’s movement towards the feeder (crossing in succession infrared lights (1) and (2)) or away from the feeder (crossing in succession infrared lights (2) and (1)) triggered the stimulation of the implanted electrode to evoke an fPSP in the mPFC recording site. The animal’s placing its head into the feeder also triggered a stimulus to evoke an fPSP (see left diagram in Figure 2B).

Phase 1 sessions were repeated daily for up to 4 times. Since the animal was maintained at 80–85% of its body weight, it presented a natural tendency to visit the feeder across the session. In Figure 2A (sessions 1–4) it is illustrated the increased confidence in collecting the available food during the No Shock phase. In Figure 2B are illustrated the relative amplitudes of fPSPs collected from the three selected synapses during the last Phase 1 session. As shown, no significant changes in fPSPs evoked at the three selected synapses [(*F*_(3,65)_ = 0.18, *P* = 0.9) for MD-mPFC, (*F*_(3,41)_ = 0.38, *P* = 0.76) for CA1-mPFC, and (*F*_(3,36)_ = 0.36, *P* = 0.78) for Amyg-mPFC] were observed in Phase 1 for any of the behaviors included in the study (i.e., fleeing from the feeder, approaching the feeder, and feeding). For comparative purposes, baseline fPSPs (computed as 100%) were always recorded just before the beginning of the experimental session.

During Phase 2 sessions (see left diagram in Figure 2C), the animal received a mild electrical shock (500 μs and 0.5–1 mA) on the infraorbital nerve each time it introduced its head into the feeder, with a delay of 500 ms, i.e., once the fPSP evoked for the crossing of the third infrared light was finished and properly recorded. The presence of a mild punishment accompanying the act of feeding did not decrease the number of times the animal approached the feeder across the five Phase 2 sessions (Figure 2A, sessions 5.9), and maintained the tendency to get some food from the dispenser. In this case, fPSPs evoked at the three selected synapses decreased significantly [(*F*_(3,63)_ = 9.34, *P* < 0.001) for MD-mPFC, (*F*_(3,49)_ = 11.57, *P* < 0.001) for CA1-mPFC, and (*F*_(3,36)_ = 3.63, *P* < 0.05) for Amyg-mPFC] in amplitude for the three behaviors included in this study (Figure 2C).

Finally, during Phase 3 sessions (see left diagram in Figure 2D), the animal received a strong shock (5 ms and 0.5–1 mA) on the infraorbital nerve when putting its head into the feeder. This strong punishment drastically decreased the number of attempts made by the experimental animals to obtain some food (Figure 2A, sessions 10–12). In addition, the presentation of a strong shock during such attempts resulted in a noticeable decrease in the amplitude of fPSPs evoked at the MD-mPFC (*F*_(3,47)_ = 5.12, *P* < 0.01) and CA1-mPFC (*F*_(3,37)_ = 5.89, *P* < 0.01) synapses during the performance of the three behaviors (fleeing from the feeder, approaching the feeder, and feeding) included in the study (Figure 2D). FPSPs evoked at the Amyg-mPFC (*F*_(3,65)_ = 0.18, *P* = 0.11) synapse also decreased in amplitude, but were significantly smaller only during fleeing (Figure 2D).

### Changes in fPSPs evoked at three different prefrontal afferents during a decision-making, operant conditioning task

As illustrated in Figure 3A, mice were trained with a fixed-ratio (FR1:1) schedule to obtain a small pellet of food each time they pressed a lever located near to the feeder. In a preliminary phase, the animal was trained in the Skinner box until reaching the initial criterion—namely, just pressing the lever 15 times per session and collecting the supplied pellet of food.

During the first phase of the operant conditioning task (Figures 3A,B), the animal was placed in the Skinner box for 20 min during four successive daily sessions. This number of sessions was enough to reach the selected criterion—i.e., to obtain a minimum of 15 pellets per session in two successive sessions (Figure 4A). The animal was stimulated at the implanted synapse when approaching (1 → 2) the lever or when fleeing (2 → 1) from it. No trigeminal shocks were applied during this phase, but the animal was also stimulated at the selected synapse when pressing the lever (3). The mean number of lever presses was 20.9 ± 2.2 during the last Phase 1 session. In the absence of any trigeminal shock, a maximum of 40 stimuli were presented to the implanted synapse of the animal during each session. Figure 3B illustrates the results collected from fPSPs evoked during Phase 1. Collected results indicate that, in comparison with baseline values, there was an increase in the amplitude of fPSPs evoked at the CA1-mPFC (*F*_(3,71)_ = 5.06, *P* < 0.01) and Amyg-mPFC (*F*_(3,54)_ = 2.79, *P* < 0.05) synapses, in contrast with a decrease in the amplitude of fPSPs evoked at the MD-mPFC (*F*_(3,150)_ = 3.93, *P* < 0.5) synapse. Baseline fPSPs were collected before the beginning of the session, but with the animal already located in the Skinner box (see Section Methods).

During the second phase of the operant conditioning task (Figures 3A,C), the experimental animal received a mild trigeminal shock (500 μs, 0.5–1 mA) 500 ms after pressing the lever. This phase consisted in five successive sessions. The presentation of an unpleasant trigeminal stimulation during lever presses decreased the rate of this behavior from the 1st session, but without producing its complete disappearance (Figure 4A). The mean number of lever presses was 12.4 ± 1.5 during the first Phase 2 session—i.e., significantly (*P* < 0.05, Bonferroni *t*-test) less than during the last Phase 1 session. The number of lever presses increased mildly during this Phase 2, reaching 13 ± 2.3 during the last Phase 2 session. In this situation, the amplitude of fPSPs evoked at the CA1-mPFC (*F*_(3,110)_ = 5.5, *P* < 0.001) changed during the execution of the three studied behaviors (fleeing, approaching and levering; Figure 3C). In contrast, no changes were observed at the MD-mPFC (*F*_(3,86)_ = 0.09, *P* = 0.9) and Amyg-mPFC (*F*_(3,123)_ = 1.6, *P* = 0.1) synapses. Thus, there was a significant decrease in the amplitude of fPSPs evoked at the CA1-mPFC synapse during lever presses and no significant changes in the other two synapses (Figure 3C, *post hoc* tests).

During the third phase of the operant conditioning task (Figures 3A,D), the experimental animal received a strong trigeminal shock (5 ms, 0.5–1 mA) 500 ms after pressing the lever. This phase was repeated for three successive sessions. The presentation of a strong trigeminal shock together with each lever press decreased the total number of lever presses until its almost complete disappearance (Figure 4A). The mean number of lever presses was 1.08 ± 0.4 during the last Phase 3 session—i.e., significantly (*P* < 0.001, Bonferroni *t*-test) fewer than the 3rd and 4rd sessions of the Phase 1. In this situation, the amplitude of fPSPs evoked at the MD-mPFC and Amyg-mPFC synapses changed during the execution of the three studied behaviors [(*F*_(4,46)_ = 6.67, *P* < 0.001) and (*F*_(4,70)_ = 3.36, *P* < 0.05), respectively] (Figure 3D). In contrast, no changes were observed at the CA1-mPFC synapse (*F*_(4,57)_ = 2.08, *P* = 0.09). In addition, there was a significant change in the amplitude of fPSPs evoked during lever presses; specifically, *post hoc* tests detected a decrease in the amplitude of fPSPs evoked at the CA1-mPFC synapse during lever presses (Figure 3F), and a significant increase in the amplitude of fPSPs evoked at the Amyg-mPFC synapse (Figure 3G). FPSP amplitudes collected with the animal located near the lever, but without pressing it, presented similar (although not significant) changes to those evoked during lever presses (see “Quasi lever” values in Figure 3D).

Although there was an evident decrease of lever presses during the 2nd and 3rd phases of the experiment (Figure 4A), there was not a concomitant decrease in food consumption, as the animals ate all the collected pellets.

Figures 3E–G illustrates changes evoked in the amplitude of fPSPs at the MD-mPFC (E), CA1-mPFC (F), and Amyg-mPFC (G) synapses for the different behaviors and experimental situations. On the whole, fPSPs evoked at the MD-mPFC synapse decreased during the successive sessions when the lever was approached, and increased when it was pressed (Figure 3E). In contrast, fPSPs evoked at the Amyg-mPFC synapse increased in amplitude when pressing the lever in presence of the strong punishment (Figure 3G). Finally, CA1-mPFC fPSPs decreased across the successive phases (from No Shock to Strong shock) with the increased punishment for the three studied behaviors (Figure 3F). Figure 4 presents the evolution of fPSPs evoked at the three selected synapses across the successive Phase 1, 2, and 3 sessions during lever presses. Here again, the different evolution of evoked fPSPs can be seen, since those evoked at the CA1-mPFC synapse decreased with increased punishment (Figure 4C), while evoked at the Amyg-mPFC synapse increased (Figure 4D), and those evoked at the MD-mPFC synapse did not present any significant change across this task (Figure 4B).

### Electrophysiological properties of the three selected synapses determined in behaving mice

An attempt was made to determine some basic electrophysiological properties of the three selected synapses in alert behaving mice, to see whether those properties could be related with the changes evoked in their fPSPs during the experimental tasks included in this study. Onset latencies and amplitudes of recorded fPSPs are indicated in Table 1.

**Table 1:** **Onset latency and amplitude averages corresponding to fPSP recorded at the medial prefrontal cortex (mPFC) after stimulation at the mediodorsal (MD) thalamus, CA1 hippocampal area and amygdala (Amyg) of three different mice**.

	fPSP onset latency (ms)	fPSP amplitude (mV)
MD-mPFC	9.12 ± 0.13	0.58 ± 0.01
CA1-mPFC	5.8 ± 0.07	0.28 ± 0.01
Amyg-mPFC	9.6 ± 0.14	0.14 ± 0.01

*Data represent mean values ± SEM, *n* = 40*.

As shown in Figures 5A–C, the three synapses presented I-O curves that increased in fPSP amplitude with the increase in stimulus intensity (0.05 to 0.5 mA), reaching similar final saturating values. However, while the increase in fPSP amplitude was—in general—steady, the I-O curve evoked at the CA1-mPFC synapse presented an early, large increase in fPSP amplitudes with smaller stimulus intensities than the other two.

The three synapses presented a significant (*P* ≤ 0.05, Student-Newman-Keuls test) P-P facilitation at low inter-pulse intervals (Figures 5D–F). Peak facilitation was at 100 ms of inter-pulse interval for the MD-mPFC synapse and at 40 ms for the other two.

Finally, LTP was evoked at the three selected synapses by an HFS session (see Section Methods). Baseline records were collected for 15 min, by stimulus presentation at a rate of 3/min. After the HFS session, stimuli were presented again at the same rate for up to 60 min. The same rate of stimulus was presented for 15 min on three additional days (Figures 5G–I).

The HFS of the MD-mPFC synapse evoked a significant (*F*_(15,251)_ = 2.6, *P* < 0.01) LTP that reached its maximum value 24 h afterwards. A similar finding (i.e., a delayed LTP) has also been reported for the synapse between the thalamic reuniens nucleus and the mPFC (Eleore et al., 2011). As already reported (Jurado-Parras et al., 2012), the HFS of the CA1-mPFC synapse presented a small, slow-building LTP that reached its maximum value 48–72 h afterwards. Finally, the HFS of the Amyg-mPFC synapse evoked a significant fast-rising (*F*_(21,218)_ = 1.92, *P* < 0.05), long-lasting LTP. The presence of a fast-rising LTP in the Amyg-mPFC synapse seems to be coherent with the noticeable changes in fPSP amplitude observed in this synapse during both shock/food and Skinner box tasks.

## Discussion

### General remarks

Decision-making involves a wide range of cerebral areas interconnected by, commonly, bidirectional synapses, of which the ones established between MD, CA1, and Amyg with the mPFC seem to be among the most-implicated (Herry et al., 1999; Thierry et al., 2000; Bechara et al., 2003; St. Onge et al., 2012). In the present study, we have analyzed the activity of the MD-mPFC, CA1-mPFC, and Amyg-mPFC synapses in alert behaving mice during the performance of two acquired tasks: a fear conditioning task, and an operant conditioning in a Skinner box, in which a decision-making factor, related to food reward, was included. The most important difference between the two tasks was that in the latter a lever press to obtain food was punished on occasions with an electrical shock, while in the former, it was the action of introducing the head into the feeder that triggered the shock. The collected results demonstrate differences between the two tasks. In the fear conditioning decision-making task, the general tendency was an inhibition of fPSPs evoked during the different phases of the experiment, more pronounced when the punishing shock was stronger (Figures 2C,D). In this regard, it has been reported that conditioned fear, *per se*, depresses prefrontal synaptic excitability (Herry and Garcia, 2002). In addition, and taking into account the role of the prefrontal cortex in cognitive functions (Bechara et al., 2003; Bechara, 2005; Floresco et al., 2008; St. Onge et al., 2012), the decrease in prefrontal synaptic transmission may be related to processing of cognitive information, such as the presence of danger. In the operant conditioning decision-making task, different tendencies were observed. Thus, during the first phase, all the synapses underwent an inhibition from fleeing to lever actions (Figure 3B). In contrast, during the second phase, no changes were observed during moving away from or approaching the lever, and lever pressing evoked no changes in the MD-mPFC and Amyg-mPFC synapses, while the CA1-mPFC synapse showed an inhibition (Figure 3B). Finally, during the third phase, the most significant observation was the high potentiation of the Amyg-mPFC synapse when the lever was pressed. At the end, comparison of the two shock phases (2 and 3) revealed a different pattern of potentiation, or inhibition, of the three synapses (mostly CA1-mPFC and Amyg-mPFC) when the lever was pressed, a reflection of the different influences of the MD, CA1, and Amyg neural sites on the activation state of mPFC circuits.

Another possible way to explain the reported findings is that, when a decision is being taken, the mPFC responds differently to the afferent volley coming from these three areas. In this regard, an increase in mPFC firing during conditioning stimulus presentations, as well as during the CS-US interval in a trace fear conditioning, has been reported (Baeg et al., 2001; Gilmartin and McEchron, 2005). This could result in a firing-pattern-dependent facilitation or depression, as has been reported in the mPFC during a working memory task (Fujisawa et al., 2008).

In any case, the collected results showed that the patterns of synaptic potentiation, or depression, when the animal carried out the action of eating the offered food during the fear conditioning task were different to those when it pressed a lever to obtain a pellet during the operant conditioning task—aware in both cases of the punishment that the act involved.

As the recording site was the same for the three studied synapses, the differences of the potentiation in these three cases during the operant conditioning decision-making task could be related not with the mPFC state, which may be the same in all the cases, but with the state of the three areas included in the present study that project to it. In other words, the origin of these changes of synaptic potentiation could be presynaptic. With regard to the fear conditioning task, the same proposal is not possible, as all the synapses are inhibited during the shock phases. Indeed, this latter task is less complex than the operant conditioning one, as the animal’s dilemma is to eat or not to eat the offered food, rather than to press or not to press the lever to obtain it. In relation with the above considerations, the input-output (I-O) curves, paired pulse facilitation (P-P), and LTP tests showed different patterns, which could have some relationships with the observed behavioral results. Thus, while the I-O curves of the MD-mPFC and Amyg-mPFC synapses increased progressively, the CA1-mPFC increased rapidly at low intensities, reaching asymptotic amplitudes at two-third of the values used in the other two synapses. This fact must be related with the soft increase in amplitude observed following HFS during the LTP experiment, as the intensities used to obtain baseline values should be enough to reach high fPSP amplitudes. Moreover, the fPSP ratio obtained for this synapse during the paired-pulse test was the higher at lower inter-stimulus intervals, suggesting that the CA1-mPFC synapse is not only the most excitable, but also the most facilitating one. In addition, it is interesting to remark the different LTP profiles collected in this study. For example, and as it has been described previously (Jurado-Parras et al., 2012), MD-mPFC and CA1-mPFC synapses showed a progressive increase in their fPSP amplitudes, reaching the bigger LTP values 24–72 h after the HFS session. In contrast, the Amyg-mPFC synapse showed the larger fPSP amplitudes just after the HFS, with a pattern similar to that described previously (Tan et al., 2010) and to the classic reports for the CA3-CA1 hippocampal synapse (Gruart et al., 2006; Jurado-Parras et al., 2012).

### Role of the MD-mPFC synapse in decision-making tasks

Since the classic definition of the prefrontal cortex made by Rose and Woolsey (1948), based on its projections to the MD thalamic nucleus, the role of MD-mPFC synapses in decision-making and goal-directed behavior tasks in primates and non-primates has been widely addressed (Uylings et al., 2003; Block et al., 2007; Izquierdo and Murray, 2010; Funahashi, 2013). Our results show a general inhibition in the shock phases of the fear conditioning task, which contrast with what is seen in the operant conditioning task when the lever is pressed, as significant differences are stabilized between phases, and between the first Phase and the baseline. Thus, the inhibition and repotentiation of the MD-mPFC pathway during the operant conditioning (Figure 3E) must be related with the consequences of lever pressing, more than with the context. In this regard, it has been shown that lesions of the MD disrupt visual stimulus/food-reward associations (Gaffan and Murray, 1990; Gaffan et al., 1993), although a role in contextual fear conditioning after post-training lesions of this area has also been reported (Li et al., 2004). The strong responses observed in MD neurons during the presentation of cues associated with a reward, but not after it (Kawagoe et al., 2007), could be related with the state in which the MD-mPFC synapse may be at the very moment the stimulus is triggered, even if the predictive stimulus (in our case, the lever) is simpler. In this situation, the recruiting of postsynaptic cells receiving continuous inputs from the MD nucleus when the animal is approaching the lever could be less likely than once the lever has been pressed (as during the strong shock phase).

### Role of the CA1-mPFC synapse in decision-making tasks

The results obtained with the activation of the CA1-mPFC synapse in the two different experimental situations indicate a progressive inhibition associated with the two shock phases during the fear conditioning task, for the three considered behaviors (fleeing, approaching, and feeding (Figure 2)). These results are in accordance with the marked inhibition in the two shock phases and the potentiation in the no-shock one (mostly for fleeing and lever presses) during the operant conditioning task (Figure 3F). The findings correlate with descriptions of the timing role of the hippocampus in cognitive processes, in the sense that for short periods of time the function of this area seems to be significant, although once the task is learned, other areas play a more significant role (Euston et al., 2012; Yu and Frank, 2014). Nevertheless, as an inhibition was found for both fleeing and lever presses during each phase of the operant conditioning, it is necessary to consider changes in the CA1-mPFC synapse that could modify its functional strength. For example and as previously reported, the spectral power of theta oscillations increases in strength during a spatial decision-making task (Belchior et al., 2014). Furthermore, during the learning periods of a novel task, or even during changes in an already acquired task, the presence of sharp-wave ripples (SWRs) is prevalent, but as soon as the animal becomes familiar with the task, the number of SWRs begins to decrease gradually (Yu and Frank, 2014).

Moreover, anatomical evidence suggests that rat CA1 hippocampal afferents collaterally innervate excitatory projecting pyramidal neurons and inhibitory interneurons, creating a disynaptic, feed-forward inhibition microcircuit in the mPFC (Takita et al., 2007). It has been proposed that, in this situation, the sequential transmission of the hippocampal-mPFC pathway can phasically drive the collateral feed-forward inhibition system through activation of GABA_A_ receptors, bringing an active signal filter to the various types of impulse train that enter the mPFC from the hippocampus (Takita et al., 2007). Thus, the different oscillation patterns and the feed-forward inhibitions may affect the functional state of hippocampal-mPFC synapses in each moment of the task—in the present case by promoting a synaptic depotentiation when a decision is being taken.

### Role of the Amyg-mPFC synapse in decision-making tasks

It is generally accepted that the hippocampus plays a primary role in the acquisition of new memories, but not so much in their retrieval. In the same sense, some authors hypothesize that the Amyg is equivalent to the hippocampus with regard to emotions, that is, necessary for acquiring new emotional attributes, but not for the retrieval of already acquired emotional states (Bechara et al., 2003). In any case, there are studies that involve the Amyg in the retrieval of appetitive conditioned responses (Gallagher et al., 1990). In the present study, the progressive inhibition of the Amyg-mPFC synapse observed along the successive phases of the fear conditioning task contrasted with the changes in synaptic strength detected during the operant conditioning task. Indeed, whereas a potentiation for the three selected behaviors was observed during the no-shock phase (similarly to the pattern observed for the CA1-mPFC pathway), in the third strong shock phase the pattern was completely opposite to the one observed in the CA1-mPFC pathway. Thus, whereas the task consisted only of learning to press a lever in order to obtain a pellet of food, the action of pressing the lever implied a mild depotentiation (as compared with values collected during approaches to the lever); when the task obliged the animal to press the lever in the presence of a strong trigeminal shock, the effect was a significantly larger potentiation of the Amyg-mPFC synapse. In this regard, it has been reported that the amygdala-prefrontal circuitry regulates effort-based decision-making, since an inactivation of the BLA impaired it, reducing the preference for high rewards that involve a large effort (Floresco et al., 2008). In this regard, Ostrander et al. (2011) have shown for a reklevant rodent cue-based effortful task that corroborated and expanded on that finding by the Flroesco group. Similar effects have been observed after the disruption of the mPFC-Amyg (but not the Amyg-mPFC) pathway during risk-based decision-making tasks (St. Onge et al., 2012), in which the punished subject increases its choices for greater, riskier, options. Thus, the mPFC-Amyg pathway should play a key role in the potentiation of the Amyg-mPFC synapse observed when the mouse decides to press the lever during the operant conditioning decision-making task in a rather risky situation (St. Onge et al., 2012)—i.e., in the phase including a strong trigeminal shock, and the firsts sessions of the mild one. In accordance with these results, the mPFC-Amyg pathway must undergo a strong inhibition, as in the disruption performed experimentally (St. Onge et al., 2012), just before the animal decides to press the lever in the presence of a contingent punishment, and, as a consequence of this, the Amyg-mPFC pathway could go through a brief facilitatory state (i.e., an Up state), similar to what has been reported for the thalamo-cortical circuits (Rigas and Castro-Alamancos, 2009). The fast generation of LTP reported here for the Amyg-mPFC synapse (in contrast with the slow LTP build-up observed for the other two synapses) could facilitate these fast changes in synaptic strength.

## Conclusions

Results collected during the fear conditioning decision making task indicate a general decrease in the strength of the evoked potentials when animals inhibited their natural appetitive behaviors, suggesting a disfacilitation or inhibition of the prefrontal cortex. On the other hand, results collected during the more complex decision-making, operant conditioning task showed different patterns in the activity-dependent strength of evoked fPSPs when the animals inhibited their learned appetitive behaviors, perhaps unmasking the different roles of each synapse in the decision making process. Then, these results suggest a significant involvement of these three synaptic inputs to the mPFC during the performance of different behaviors related to specific decision-making tasks.

## Conflict of interest statement

The authors declare that the research was conducted in the absence of any commercial or financial relationships that could be construed as a potential conflict of interest.
